# Association of chondroadherin with leiomyosarcoma

**DOI:** 10.1016/j.gore.2023.101144

**Published:** 2023-02-06

**Authors:** Sefik Gokce, Dilsad Herkiloglu, Ecmel İsik Kaygusuz, Ozge Cevik, Sarfraz Ahmad

**Affiliations:** a Department of Obstetrics and Gynecology, Yeni Yuzyil University School of Medicine, İstanbul, Turkey; b Pathology Department, Zeynep Kamil Training and Research Hospital, İstanbul, Turkey; c Aydin Adnan Menderes University, School of Medicine, Department of Biochemistry, Aydin, Turkey; d AdventHealth Medical Center, Orlando, FL 32804, USA

**Keywords:** Leiomyosarcoma, Chondroadherin, Myoma

## Abstract

The leiomyosarcoma (LMS) subtype of uterine sarcoma accounts for 1–2 % of uterine neoplasia cases. The present study aimed to demonstrate that the gene and protein chondroadherin (CHAD) levels may serve as novel biomarkers for predicting prognosis and devising novel treatment models for LMS. A total of 12 patients diagnosed with LMS and 13 patients diagnosed with myomas were included in the study. The extent of tumour cell necrosis, cellularity and atypia and the mitotic index of each patient with LMS were determined. CHAD gene expression was significantly increased in cancerous tissues compared with that in fibroid tissues (2.17 ± 0.88 vs 3.19 ± 1.61; P = 0.047). The mean CHAD protein expression in tissues was higher in LMS cases but this was not statistically significant (217.38 ± 93.9 vs 177.13 ± 66.67;P = 0.226). Positive significant correlations were obtained between CHAD gene expression and mitotic index (r = 0.476; P = 0.008), tumour size (r = 0.385; P = 0.029) and necrosis (r = 0.455; P = 0.011). Furthermore, there were significant positive correlations between CHAD protein expression levels and tumour size (r = 0.360; P = 0.039) and necrosis (r = 0.377; P = 0.032). The present study was the first to demonstrate the significance of CHAD in LMS. The results suggested that, due to its association with LMS, CHAD has predictive value in determining the prognosis of patients with LMS.

## Highlights

• CHAD gene expression was significantly increased in cancerous tissues compared with that in fibroid tissues.
• Positive significant correlations were obtained between CHAD gene expression and mitotic index, tumour size and necrosis.
• Furthermore, there were significant positive correlations between CHAD protein expression levels and tumour size and necrosis.
• The present study is the first to demonstrate the significance of CHAD in LMS.
• The results suggested that, CHAD has predictive value in determining the prognosis of patients with LMS.

## 1 Introduction

Leiomyomas are the most common tumour type in females and are observed in 70 % of cases of premenopausal females (Cramer and Patel, 1990, Arslan et al., 2005). Although myomas are known to be benign, they may cause serious morbidity in patients depending on their size, number and location (Bulun, 2013). In some cases, fibroids can develop into the leiomyosarcoma (LMS); for example, if it grows fast, if it is detected in the advanced age group cases. LMS subtype of uterine sarcoma makes up 1–2 % of uterine neoplastic tumours (Roberts et al., 2018). Following the standard treatment of surgical operation, the 5-year survival rate of patients with LMS is only about 25 % due to the aggressive behaviour of the cancer (Wu et al., 2006, Kapp et al., 2008). It is important to make an accurate and early diagnosis of uterine LMS (uLMS), which cannot be distinguished from fibroids through imaging and clinical examinations. Various agents, including molecular gene-targeted therapies, have been evaluated for this type of cancer and for other gynaecologic malignancies; however, they have had limited efficacy (Kapp et al., 2008). The current lack of understanding of the mechanisms underlying the tumorigenesis of uLMS appears to be the main obstacle in the development of novel therapeutic targets. There is a requirement for early diagnosis and novel treatment modalities that may support current approaches to improving survival outcomes for patients with uLMS.

Chondroadherin (CHAD) is a matrix protein that mediates cell adhesion by regulating the cytoskeletal structure of chondrocytes interacting with each other (Deng et al., 2017, Larsson et al., 1991). Pathways linked to CHAD expression are associated with focal adhesion, extracellular matrix (ECM) protein receptor interaction and the regulation of the actin cytoskeleton (Deng et al., 2017). However, reports on other functions of CHAD, including its role during carcinogenesis and cancer development remain limited.

Therefore, the present study assessed the relationship between uLMS and CHAD and examined whether CHAD may be used as a marker in the staging process of uLMS cases and as a valuable prognostic factor.

## 2 Patients and methods

*Patients*. The present study included consecutive samples from 12 patients that were diagnosed as ‘LMS’ after histopathological examination and from 13 patients that were diagnosed as ‘myoma’ at our tertiary care hospital (Zeynep Kamil Training and Research Hospital, Istanbul, Turkey) between March 2010 and April 2020. Detailed pathology reports and clinical information on the cases were obtained through archive scanning. Patients with pathological diagnoses of malignant mixed mullerian tumour, endometrial stromal sarcoma, and endometrial cancer and those who had received chemotherapy or radiotherapy were not included in the study. As a result of the information obtained, it was observed that patients either underwent myomectomy or hysterectomy and bilateral salpingo-oophorectomy, as well as pelvic and/or *para*-aortic lymphadenectomy operations according to the frozen examination results. From the samples of each patient diagnosed with LMS, tumour cell necrosis, cellularity and atypia were determined, and the mitotic index was calculated, and staging was performed using the 2009 International Federation of Gynaecology and Obstetrics (FIGO) classification system (Prat, 2009). In addition, pathological diagnoses were made according to the World Health Organization 2019 classification. Tumor preparations of the cases from the pathology archive were re-evaluated and CHAD gene expression and protein levels were examined in appropriate blocks using ELISA and PCR test methods.

*Gene expression of CHAD by reverse transcription-quantitative (RT-q)PCR.* From each paraffin block, five tissue sections (each 10-μm thick) were collected into 1.5-mL microfuge tubes. RNA isolation from tissues was performed in duplicate through the use of a commercially available FFPE RNA isolation kit (Cat. No. K156002; Invitrogen; Thermo Fisher Scientific, Inc.). A total of 1 µg RNA was reverse transcribed using a High Capacity cDNA Reverse Transcription Kit (Applied Biosytems; Thermo Fisher Scientific, Inc.) according to the manufacturer’s protocols. Primer sequences were as follows: CHAD forward, 5′-TCCACCGGAGAGACTACTGA-3′ and reverse, 5′-GGACGTGTCTAGGTGTAGGC-3′; and GAPDH forward, 5′-AGGGCTGCTTTTAACTCTGGT-3′ and reverse, 5′-CCCCACTTGATTTTGGAGGGA-3′ (Millipore-Sigma). A total of 100 ng of cDNA was amplified using SYBRGreen PCR Master Mix (Applied Biosytems; Thermo Fisher Scientific, Inc.) on the ABI StepOne Plus detection system (Applied Biosystems; Thermo Fisher Scientific, Inc.) using the following program: 95˚C for 10 min and then 40 cycles of 95˚C for 15 *sec* and 60˚C for 1 min. Gene expression was calculated using the comparative 2^-ΔΔCq^ method with StepOne software version 2.3 (Applied Biosystems; Thermo Fisher Scientific, Inc.) and normalized to the corresponding GAPDH levels (Schmittgen and Livak, 2008). Data were expressed as fold induction relative to the control values (Acidereli et al., 2021).

*Protein levels of CHAD by ELISA*. A total of four formalin-fixed paraffin-embedded tissue sections (each 10–15 μm thick) were collected into a 1.5-mL centrifuge tube. Samples were incubated with 250 μL buffer (pH 7.5; 0.05 M Tris, 1 mM EDTA and 0.5 % Tween-20). Protein extraction from all samples was performed as previously described (Nicholson et al., 2011). Protein concentrations were measured with the Bradford method and presented as μg/mL. (Bradford, 1976). The CHAD levels were measured with sandwich ELISA in accordance with the manufacturer’s protocols (Cat. No. EH1724; Fine Test) with an inter-assay coefficient of variation (CV) of < 12 % and an intra-assay CV of < 10 %, respectively. Mean minimum detectable quantity of human CHAD was 46.875 pg/mL. CHAD levels were determined as the protein concentration and data were presented as pg/μg protein. All ELISA measurements were performed using a microplate reader (BioTek Epoch; BioTek Instruments, Inc.).

*Histopathologic evaluation*. After fixation, samples were embedded in paraffin blocks and cut into 5-μm-thick sections using a Leica RM2125RTS microtome device (Leica Biosystems). Selected paraffin sections were subjected to H&E staining for morphological evaluation. All slides were examined under a light microscope (Olympus BX-51; Olympus Corporation).

*Statistical analysis*. Values are expressed as the mean ± standard deviation (SD) from at least three independent experiments. One-way analysis of variance (ANOVA) was applied to evaluate the differences among the multiple groups. Student’s *t*-test was used to perform statistical comparisons between two groups. If the variances were homogeneous, the two groups were compared using the least-significant difference method. Otherwise, Dunnett’s T3 method was included to analyse non-homogeneous variances between the two groups. Spearman correlation coefficient analysis was performed to assess the correlation between variable levels of CHAD protein and gene expression with the stage and differentiation of LMS. All statistical tests were two-sided and *p* < 0.05 was considered to indicate a significant difference. All analyses were performed using SPSS version 18.0 (SPSS, Inc.).

## 3 Results

*Patient characteristics.* According to the FIGO classification, four patients diagnosed with LMS were at stage 1a, six patients were at stage 1b, one patient was at stage 2a, and one patient was at stage 2b. The patient and the control groups were similar in terms of their mean age (54.3 ± 8.9 vs 52.4 ± 9.1 years; *p* = 0.587). Demographics and clinical characteristics of the participants are presented in Table 1.

**Table 1:** Demographics and clinical characteristics of the participants.

Parameter	Leiomyosarcoma (n = 12) Mean ± SD or n	Myoma (n = 13) Mean ± SD or n	*p*-value
Age, years	54.3 ± 8.9	52.4 ± 9.1	0.587
Body height, cm	162.8 ± 6.0	161.4 ± 6.6	0.585
Body weight, kg	64.8 ± 13.5	63.5 ± 12.1	0.802
BMI, kg/m^2^	24.4 ± 4.7	24.6 ± 5.3	0.922
Operation			
Myomectomy	3	13	
TAH + BSO	4	0	
TAH + BSO + PPLND	5	0	
Stage			
1a	2	0	
1b	8	0	
2a	1	0	
2b	1	0	
Complications	0	0	
Tumour size, cm	10.2 ± 8.5	8.8 ± 7.5	0.755

Abbreviations: SD, standard deviation; BMI, body mass index; TAH, total abdominal hysterectomy; BSO, bilateral salpingo-ooferectomy; PPLND, pelvic and *para*-aortic lymph node dissection.

*CHAD levels.* The CHAD protein was mainly localised in the LMS cells and CHAD gene expression was significantly increased in cancerous tissues compared to fibroid tissues (3.19 ± 1.61 vs 2.17 ± 0.88; *p* = 0.047). Although the mean protein level in CHAD tissues was relatively higher in LMS cases, the difference was not significant (217.38 ± 93.89 vs 177.13 ± 66.67 pg/μg protein; *p* = 0.226; Table 2).

**Table 2:** Tissue chondoadherin gene and protein expression levels in patients from different groups.

Biomarker, group	N	Expression value	*p*-value
CHAD/GAPDH			0.047
Control group	13	2.17 ± 0.87	
LMS	12	3.19 ± 1.60	
CHAD, pg/µg protein			0.226
Control group	13	177.13 ± 66.67	
LMS	12	217.38 ± 93.89	

Expression values are presented as the mean ± standard deviation. *Abbreviations:* N, number; CHAD, chondroadherin; LMS, leiomyosarcoma.; GAPDH, glyceraldehyde-3-phosphate dehydrogenase.

**Fig. 1:**
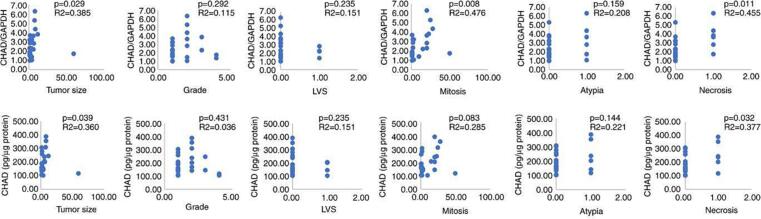
Correlation of chondroadherin gene expression and protein levels with tumor size, grade, LVS, mitosis, atypia, and necrosis. *Abbreviations:* LVS, lymphovascular space invasion; CHAD, chondroadherin; GAPDH, glyceraldehyde-3-phosphate dehydrogenase.

Positive significant correlations were obtained between the CHAD gene expression levels and mitotic index (*p* = 0.008; r = 0.476), tumour size (*p* = 0.029; r = 0.385), and necrosis (*p* = 0.011; r = 0.455). There were significant positive correlations between the CHAD protein expression levels and tumour size (*p* = 0.039; r = 0.360), and necrosis (*p* = 0.032; r = 0.377) (Table 3, Fig. 1). A positive correlation was also observed between the CHADL/GAPDH gene expression and CHADL protein expression (Table 4).

**Table 3:** Correlation between International Federation of Gynecology and Obstetrics (FIGO) classification system parameters and CHAD gene and protein expression in patients.

Item	Parameter	CHAD protein	CHAD/GAPDH
Tumor size	r	0.360	0.385
	*p*-value	0.039	0.029
Grade	r	0.036	0.115
	*p*-value	0.431	0.292
LVS	r	−0.151	−0.151
	*p*-value	0.235	0.235
Mitosis	r	0.285	0.476
	*p*-value	0.083	0.008
Atypia	r	0.221	0.208
	*p*-value	0.144	0.159
Necrosis	r	0.377	0.455
	*p*-value	0.032	0.011

*Abbreviations:* r, Pearson correlation coefficient; CHAD, chondroadherin; LVS, lymphovascular invasion;.

**Table 4:** Correlation between CHAD/GAPDH gene expression and CHAD protein expression.

Control Variables			CHAD/GAPDH	CHAD (pg/μg protein)
TISSUE TYPE	CHAD/GAPDH	Correlation	1.000	0.698
		Significance (1-tailed)		0.000
		df	0	22
	CHAD (pg/μg protein)	Correlation	0.698	1.000
		Significance (1-tailed)	0.000	
		df	22	0

Abbreviations: CHAD, chondroadherin; GAPDH, glyceraldehyde-3-phosphate dehydrogenase; df, degrees of freedom.

## 4 Discussion

CHAD, a member of proteoglycans, is a short leucine-rich repeat protein with powerful effects and key roles in cellular signalling pathways (Moreth et al., 2012). Recent research and data have determined that these proteins regulate biological functions in numerous tissue types, such as the skin, tendon, kidney, liver, and heart (Yamaguchi et al., 1990). A *meta*-analysis indicated that CHAD provides signal transmission from the ECM to the cells, with transmembrane receptors involved in the binding of the cells to the ECM, mostly as a result of interactions with integrins (Iozzo and Schaefer, 2010). As a result of these studies, the relationship with integrins in the carcinogenesis mechanism(s) and progression of CHAD and its changing cell formation have been highlighted. The role of integrins in the activation of transforming growth factor (TGF) pathology in the process of inflammation and neoplastic invasion has been demonstrated (Munger et al., 1999). Furthermore, information on the relationship between integrins and receptor tyrosine kinase has formed a basis for novel approaches to cancer treatments (Tawbi et al., 2017). In the present study, it is demonstrated that CHAD may be associated with numerous late-stage sarcomas and interacts with integrins in benign myoma and LMS tissue. Although the mean protein level in CHAD tissues was relatively higher in LMS cases, the difference was not significant. To the best of our knowledge, the present study is the first attempt to detect CHAD levels in LMS cases.

Information on the expression levels of CHAD in numerous tissue types, including the cornea, retina, Purkinje cells of the cerebellum, peripheral nerves, blood vessels in various tissues, and pancreatic islets and ovaries, have been reported (Tasheva et al., 2004). CHAD has been indicated to be prominently expressed in the cartilage of the growth plate, particularly in the proliferative and hypertrophic regions (Shen et al., (Pt 1):549–557, 1998.). The protein is a member of the leucine-rich repeat family but distinguishes itself by having a double disulphide ring near the C-terminus, whereas others have a single ring in this region. It is also unique among these proteins in that it lacks post-translational glycosylation. It does not contain the *N*-terminal extension common in other proteins (Neame et al., 1994). CHAD binds to two separate triple-helix collagen regions and to the α2β1 integrin (Mansson et al., 2001, Camper et al., 1997) on the cell surface of chondrocytes. Cells that adhere to CHAD via α2β1 take a round shape, similar to cells that adhere to the integrin binding site of fibronectin via α5β1 (Howe et al., 2002). With this shape, it provides signals that cause the spreading and formation of focal adhesions and stress induced between the cells. Cell proliferation may be induced as a result of CHAD and integrin cooperation (Camper et al., 1997). The results of the present study highlight the role of CHAD in inflammation and cancer invasion. The data of the present study confirmed our guiding hypothesis that the level of CHAD gene expression is significantly increased in LMS compared to myoma tissue.

Results from previous studies suggest that CHAD is a valid prognostic biomarker for different solid tumor types, including gynaecologic malignancies (Deng et al., 2017, Wei et al., 2020), but no peer-reviewed data had been previously published for uLMS, to the best of our knowledge. The active role of CHAD as an ECM protein in intercellular communication and invasion (Mansson et al., 2001, Camper et al., 1997) reflects the extent of malignant cell turnover and cell cycle progression induced by tumor aggression and biological behaviour.

The small number of cases in the present study may represent a limitation. However, the cases were collected over a certain time period. As is known, LMS is among the rare uterine cancers, but according to the power analysis, the results were calculated as having 80 % accuracy. For these reasons, the study was performed with this number of patients. Another limitation of the study was that it was not possible for us to obtain sufficient information about the survival and progression of the disease in the patients due to the delays in their follow-up. Therefore, there is a requirement for large-scale studies on the subject, perhaps through multi-center collaboration.

In conclusion, the present study is the first attempt to indicate the association of the level of CHAD in patients with uLMS with unfavourable poor prognosis. According to the results of the present study, CHAD, as a novel marker for uLMS, a disease with rapid progression and resistance to treatment, is able to predict the prognosis for uLMS.

Funding.

No funding was received.

Ethics approval and consent to participate.

The study was approved by the Ethics Committee of Zeynep Kamil Women’s and Children’s Diseases Training and Research Hospital (Istanbul, Turkey; approval no. 2021/115). All patients consented to the treatment according to the institutional guidelines for informed consent and all patients consented to the anonymous evaluation and analysis of their data and treatment outcomes. This study was carried out in accordance with the Declaration of Helsinki and the guidelines of the Ethics Committee of Zeynep Kamil Women’s and Children’s Diseases Training and Research Hospital (Istanbul, Turkey).

Patient consent for publication.

Not applicable.

## Declaration of Competing Interest

The authors declare that they have no known competing financial interests or personal relationships that could have appeared to influence the work reported in this paper.
